# TPP Combined with DGUC as an Economic and Universal Process for Large-Scale Purification of AAV Vectors

**DOI:** 10.1016/j.omtm.2019.11.009

**Published:** 2019-11-22

**Authors:** Zhe Yu, Siyun Zhou, Ningguang Luo, Ching Yi Ho, Min Chen, Haifeng Chen

**Affiliations:** 1 Virovek, 22429 Hesperian Boulevard, Hayward, CA 94541, USA

**Keywords:** AAV purification, three-phase partitioning, TPP, baculovirus, gene therapy, density gradient ultracentrifugation

## Abstract

Adeno-associated virus (AAV) vectors have been commonly purified through density gradient ultracentrifugation (DGUC) or column chromatography methods. Although the DGUC method can efficiently separate the empty from the full virus particles, its application in large-scale AAV purification is hindered due to its limitation in volume of each centrifuge tube. Alternatively, column chromatography is serotype-dependent, expensive, and complicated, which co-purifies both empty and full virus particles. In this study, we describe an economical and universal process using three-phase partitioning (TPP) combined with DGUC to purify large quantities of AAV vectors. First, TPP is used to remove up to 90% of the cellular impurities in the cell lysate and at the same time condense the AAV vectors into ∼10% of their original lysate volume. Second, two rounds of DGUC are employed to separate the empty from the full virus particles and at the same time remove the remaining cellular impurities. This combined process increases the capacity of ultracentrifugation by a factor of 5- to 10-fold depending on the yields of AAV serotypes. A variety of AAV serotypes such as AAV2, AAV5, AAV6, AAV9, and AAVDJ have been successfully purified with this process. Both *in vitro* and *in vivo* studies demonstrate that TPP has no detrimental impact on AAV infectivity. In a proof of concept, we performed several purification runs ranging from 3 to 25 L of Sf9 culture volume. We were able to purify more than 3e+15 viral genomes (vg) of AAV vectors from 3 L of cell culture volume with just two SW28 centrifuge tubes in a Beckman Coulter ultracentrifuge. Our data indicate that this TPP-DGUC process is economic, universal, and can be used to purify a large quantity of AAV vectors for clinical applications with just a few ultracentrifuges.

## Introduction

Recombinant adeno-associated virus (AAV) vector has emerged as one of the most versatile and successful gene therapy delivery vehicles. A number of clinical trials successfully commenced recently,^1^, ^2^, ^3^, ^4^ and patients diagnosed with lipoprotein lipase deficiency now have an option to be treated with Glybera, the first AAV1-based drug to win the regulatory approval of the European Commission.^5^ Adding to this exciting news is the approval of Luxturna by the US Food and Drug Administration (FDA), the AAV2-based drug to treat hereditary blindness.^6^ However, even though the industry is poised for the expansion into several application areas represented by orphan diseases, a simple and scalable AAV purification process is still lacking. AAV purification with the traditional method through density gradient ultracentrifugation (DGUC) using either cesium chloride (CsCl) or iodixanol media is limited by the volume each centrifuge tube can hold. For a swing bucket SW28 rotor, each centrifuge tube has a maximum volume of 38.5 mL. After adding the step gradient CsCl media, each tube can only hold ∼23 mL of lysate prepared from a cell pellet collected from 200 to 300 mL of cell culture. A 25-L production run would require 83 centrifuge tubes, or 14 ultracentrifuges, which is expensive and not practical. Although AAV purification with column chromatography is a preferred method in the industry, the process of column chromatography is serotype-dependent, expensive, and complicated, and both empty and full AAV virus particles are usually co-purified. In addition, the AAV binding capacity of the media is generally in the range of 1e+12 viral genomes (vg)/mL, which translates into the requirement of a 100-L column for purification of 1e+17 vg of AAV vectors, and the cost of such a large column is in the millions of US dollars. It has been reported that polyethylene glycol (PEG)/aqueous two-phase partitioning methods could be used to purify AAV vectors, but the method yielded limited amounts of AAV vectors and the purities were not satisfactory.^7^

Three-phase partitioning (TPP) is an emerging non-chromatographic and economical technology for the separation of bioactive molecules from natural sources.^8^ Recently, it has been used as a scalable method for purification of several proteins.^9^, ^10^, ^11^, ^12^ The principle of TPP is based on the use of *tert*-butanol and ammonium sulfate to precipitate proteins from aqueous solution. Tertiary butanol is normally completely miscible with water, but upon addition of enough salt, such as ammonium sulfate, the solution separates into two phases, a lower aqueous phase and an upper *t*-butanol phase. If protein is present in the original aqueous phase, it may, depending on the concentration of ammonium sulfate added, separate into a third phase intermediate between the lower aqueous and upper *t*-butanol phases. Although proteins can be purified by TPP, there has been no report so far that virus particles such as AAV can be purified using this process.

In the search for an economical and scalable technology for AAV purification, we decided to test the possibility of using TPP as an upstream process for AAV purification. Our goal was to combine the TPP process with CsCl ultracentrifugation so that an economic and universal process could be established. After many failed attempts, we finally developed a TPP process that can remove up to 90% of cellular impurities in an easily scalable setting, and it greatly decreases the volume of AAV samples for the downstream purification processes. Herein, we described the details of the TPP process combined with DGUC with CsCl media for large-scale AAV purification. This TPP-DGUC process not only separates the empty from the full AAV particles, but it also increases the capacity of AAV purification with ultracentrifuges by a factor of 5–10 depending on the AAV yield. This novel process can be used as an economical and universal process for large-scale AAV purification.

## Results

### Optimization of Ammonium Sulfate Concentration in the First Round of TPP

**Figure 1:**
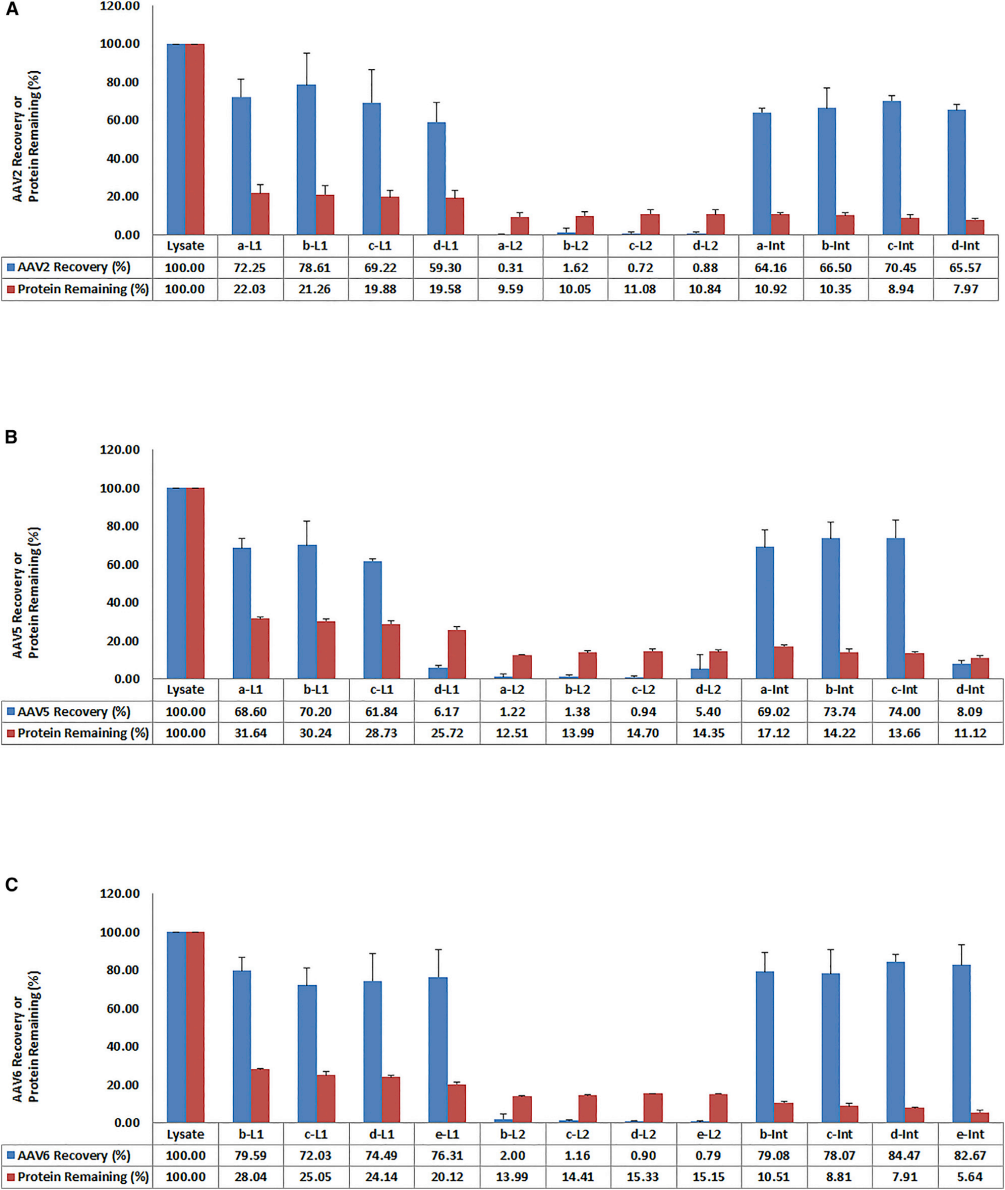
Effects of Different Concentrations of Ammonium Sulfate on Recovery of Three Serotypes of AAV Particles in the First Round of TPP (A–C) Cell lysates with (a) 10%, (b) 15%, (c) 20%, (d) 25%, and (e) 30% concentrations of ammonium sulfate, respectively, in the first round of TPP. L1, aqueous phase after first round of TPP; L2, aqueous phase after second round of TPP at fixed 35% concentration of ammonium sulfate; Int, interphase after second round of TPP at fixed 35% concentration of ammonium sulfate. Error bar indicates the SD. (A) AAV2. (B) AAV5. (C) AAV6.

In order to determine the optimal concentration of ammonium sulfate to salt-out AAV for purification, we tested a series of ammonium sulfate concentrations from 10% to 45%. However, AAV particles either remained in the lower aqueous phase at ammonium sulfate concentrations from 10% to 25% or salted-out together with cellular proteins from 30% to 45%. We reasoned that two rounds of TPP would be needed to separate AAV particles from cellular proteins and impurities. In order to further optimize the separation conditions, we performed more detailed experiments to pinpoint the suitable concentration of ammonium sulfate in each round of TPP. Since different serotypes of AAV vectors have different capsid compositions and characteristics, we chose three AAV serotypes, AAV2, AAV5, and AAV6, in our studies to test whether they would behave differently in the TPP process. Cell lysates containing these three AAV vectors, respectively, were subjected to a first round of TPP at different concentrations of ammonium sulfate, and then the first lower aqueous phase (L1) containing these AAV vectors, respectively, was subjected to a second round of TPP with a fixed 35% saturation of ammonium sulfate. The results are shown in Figures 1A–1C. All data are representative results from three independent experiments with SD indicated. As these results show, most AAV vectors remained in the L1 phase when 10% (a-L1), 15% (b-L1), or 20% (c-L1) ammonium sulfate was used. However, when the concentration of ammonium sulfate was increased to 25% (d-L1), there was some decrease of AAV2 vector recovery in the L1 phase (Figure 1A; compare c-L1 [69.22%] with d-L1 [59.30%] and c-interphase [Int] [70.45%] with d-Int [65.57%]) and a significant decrease of AAV5 vector recovery in the L1 phase (Figure 1B; compare c-L1 [61.84%] with d-L1 [6.17%] and c-Int [74.00%] with d-Int [8.09%]). Surprisingly for AAV6 vectors, even when the concentration of ammonium sulfate was increased to 30% (e-L1), there was no decrease of AAV6 vectors in the L1phase (Figure 1C; compare d-L1 [74.49%] with e-L1 [76.31%] and d-Int, [84.47%] with e-Int [82.67%]). There were nearly no AAV vectors left in the second lower aqueous phase (L2) phase (2% or less) for all three AAV serotypes tested.

We also performed protein assays to monitor the removal of cellular proteins through the whole TPP process. The results from Figures 1A–1C indicate that from 70% to 80% of cellular proteins were removed in the first round of TPP. There is a slight increase of protein-removal power when the concentration of ammonium sulfate was increased from 10% to 30%. Based on these results, 20% ammonium sulfate in the first round of TPP appeared to be optimal for all three AAV serotypes to keep AAV particles in the solution but remove most cellular proteins.

### Optimization of Ammonium Sulfate Concentration in the Second Round of TPP

**Figure 2:**
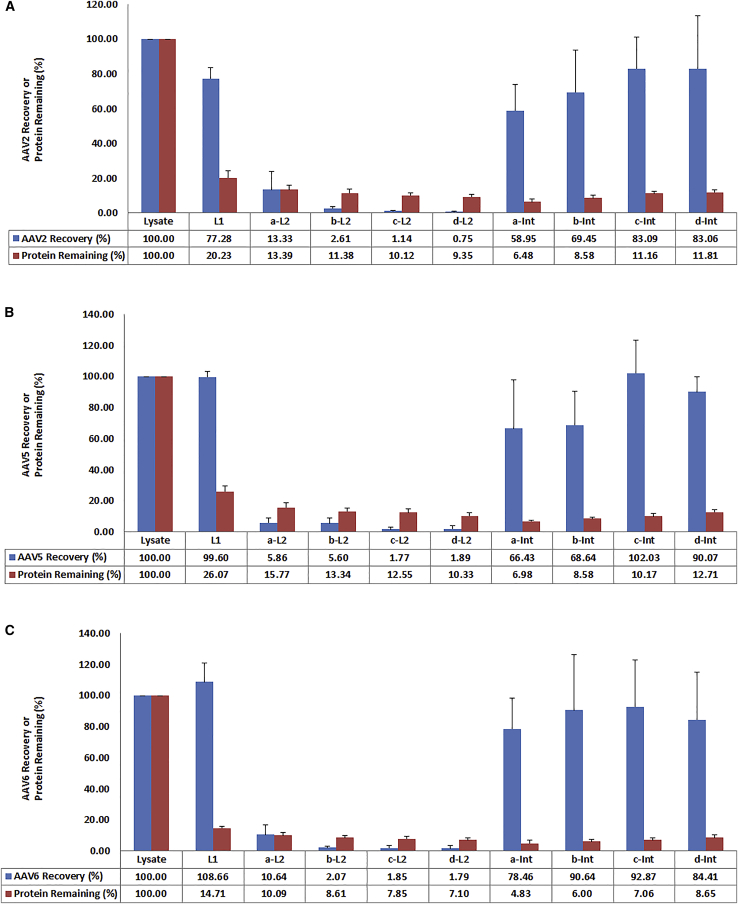
Effects of Different Concentrations of Ammonium Sulfate on Recovery of Three Serotypes of AAV Particles in the Second Round of TPP (A–C) L1, aqueous phase after first round of TPP at fixed 20% concentration of ammonium sulfate; a, b, c, and d, aqueous phase L1 divided into four parts and adjusted to contain 25%, 30%, 35%, and 40% concentration of ammonium sulfate, respectively, in the second round of TPP; L2, aqueous phase after second round of TPP; Int, interphase after second round of TPP. Error bar indicates the SD. (A) AAV2. (B) AAV5. (C) AAV6.

After the optimal concentration of ammonium sulfate in the first round of TPP was determined, we went further to test out the optimal concentration of ammonium sulfate for the second round of TPP. Cell lysates containing AAV2, AAV5, and AAV6 vectors, respectively, were subjected to a first round of TPP with fixed 20% of ammonium sulfate. The collected L1 phase of each AAV sample was divided into four equal parts and subjected to a second round of TPP with 25% (a-L2), 30% (b-L2), 35% (c-L2), and 40% (d-L2) of ammonium sulfate. The L2 and interphase of each AAV sample were collected, and AAV titer as well as protein concentration were determined. The results are shown in Figures 2A–2C. Substantial AAV recovery was observed for all concentrations of ammonium sulfate tested. Among the four different concentration values, 35% saturation shows the best recovery rate for all three AAV samples in the interphase (Figures 2A–2C, c-Int).

### Optimization of pH Value in the Cell Lysate for TPP

**Figure 3:**
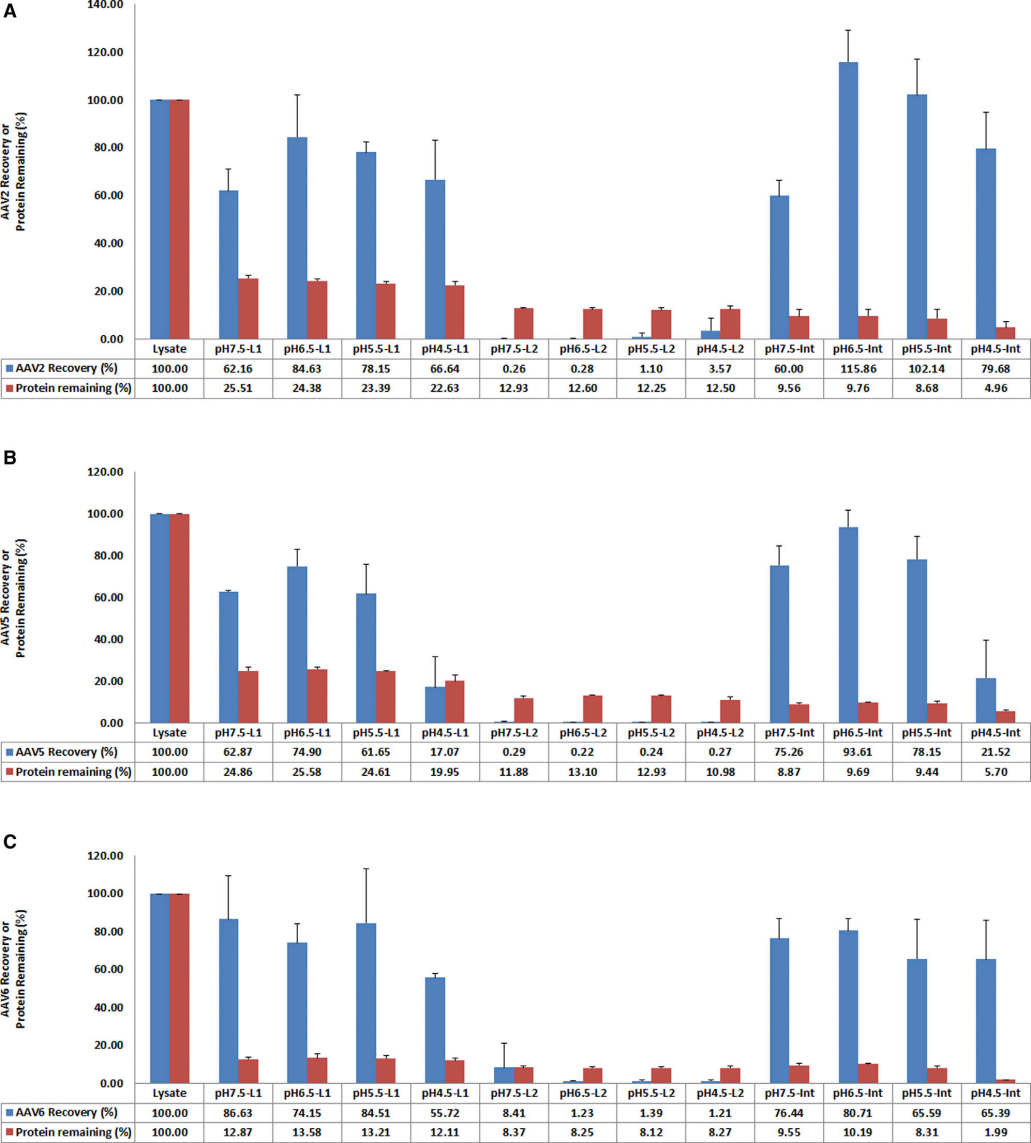
Effects of Different pH Values on Recovery of Three Serotypes of AAV Particles in the First Round of TPP (A–C) Cell lysates were divided into four parts and adjusted to pH 7.5, pH 6.5, pH 5.5, and pH 4.5 and subjected to the first round of TPP. L1, aqueous phase after first round of TPP at fixed 20% concentration of ammonium sulfate; L2, aqueous phase after second round of TPP at fixed 35% concentration of ammonium sulfate; Int, interphase after second round of TPP at fixed 35% concentration of ammonium sulfate. Error bar indicates the SD. (A) AAV2. (B) AAV5. (C) AAV6.

After the optimal saturation conditions of ammonium sulfate in the first and second TPP were determined, we decided to optimize the pH conditions in the cell lysate for AAV purification. Cell lysates containing AAV2, AAV5, and AAV6 vectors, respectively, were prepared in Sf9 lysis buffer (pH 7.5). After incubation with Benzonase for 1 h at 37°C to digest the cellular nucleic acids, ammonium sulfate was added to 20% concentration and the lysates were divided into four equal parts. Three parts of the lysates were adjusted to pH 6.5, pH 5.5, and pH 4.5, respectively, with acidic acid. All of the lysates after pH adjustments were subjected to first and second rounds of TPP and AAV titers were determined. The results are shown in Figures 3A–3C. For AAV2 and AAV6 samples, most AAV vectors were recovered in the interphase in all pH values tested, but pH 6.5 shows the best AAV recovery rate (Figures 3A–3C, pH6.5-Int). For AAV5, most AAV vectors were recovered from pH 7.5, pH 6.5, and pH 5.5. However, nearly 80% of AAV5 vectors were lost when the pH value of lysate decreased to 4.5 (Figure 3B, pH4.5-Int).

We also performed protein assays to monitor the cellular protein removal at different pH values. The results indicate that with the decrease in pH value, more and more cellular proteins were removed. For AAV2 and AAV6, more than 95% of cellular proteins were removed from the AAV samples when the cell lysates were adjusted to pH 4.5 (Figures 3A and 3C, pH4.5-Int). For AAV5, however, the removal of cellular proteins resulted in significant loss of AAV5 vectors when pH was decreased to 4.5 (Figure 3B, pH4.5-Int).

**Figure 4:**
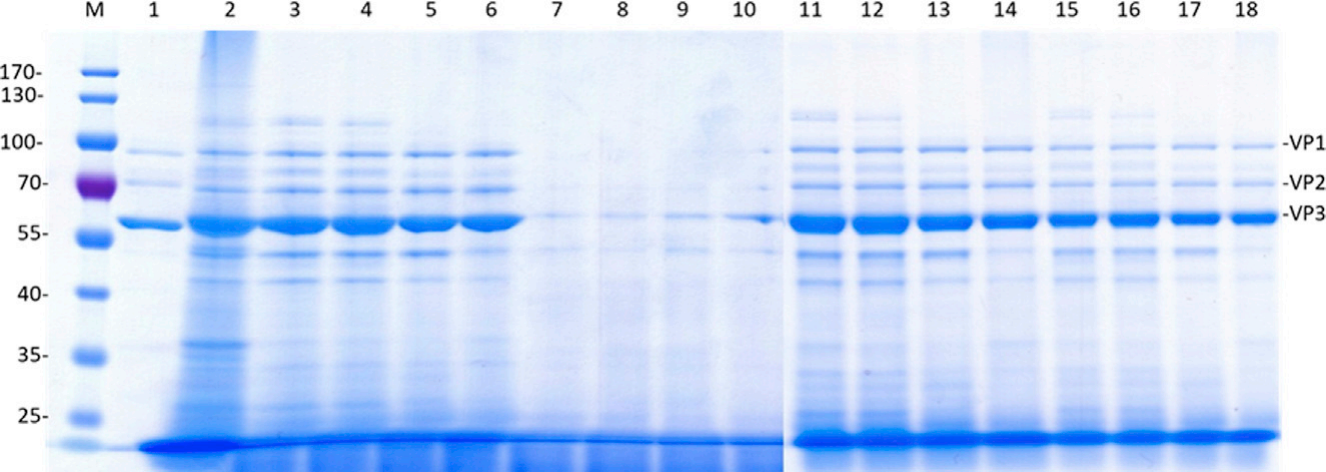
SDS-PAGE Analysis of AAV2 Samples Collected from the TPP Process with Different pH Values Samples from each step of the two rounds of TPP were collect and subjected to SDS-PAGE. The gels were stained with a SimplyBlue staining kit to show the protein bands. M, protein ladders; lane 1, purified AAV9 vector as control (1e+11 vg loaded/lane); lane 2, cell lysate; lanes 3–6, aqueous phases from first round of TPP at pH 7.5, pH 6.5, pH 5.5, and pH 4.5; lanes 7–10, aqueous phases from second round of TPP at pH 7.5, pH 6.5, pH 5.5, and pH 4.5; lanes 11–14, interphases at pH 7.5, pH 6.5, pH 5.5, and pH 4.5 after second round of TPP; lanes 15–18, same samples as lanes 11–14 but with 50% fewer AAV2 vectors loaded.

We performed SDS-PAGE analysis to monitor the purification process at different pH values with the TPP method, and a representative SDS-PAGE image is shown in Figure 4. From the gel image we can see that after two rounds of TPP, most cellular proteins were removed and clear AAV2 capsid proteins VP1, VP2, and VP3 were observed in all pH values tested, but pH 4.5 showed the best cellular protein removal from the AAV sample (Figure 4, fractions 15–18).

### Large-Scale Purification of AAV Vectors with TPP followed by DGUC

**Figure 5:**
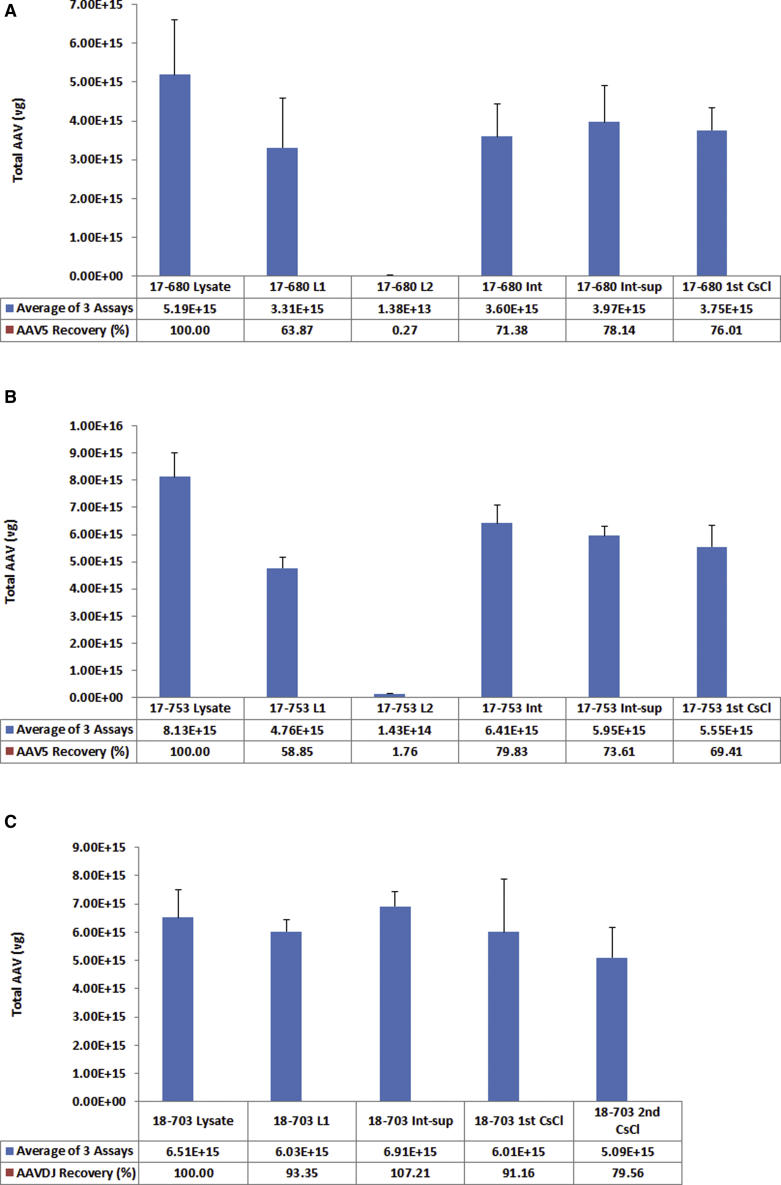
Large-Scale Purification of Three Batches of AAV Vectors (A–C) Cell lysates from three different volumes of Sf9 cell culture double-infected with 10 MOIs of rBV carrying rep and cap genes and 5 MOIs of rBV carrying gene of interest (GOI) were subjected to the TPP process followed by one or two rounds of CsCl ultracentrifugation. L1, aqueous phase after first round of TPP at 20% concentration of ammonium sulfate; L2, aqueous phase after second round of TPP at 35% concentration of ammonium sulfate; Int, interphase after second round of TPP; Int-sup, supernatant harvested after centrifugation of Int at 8,000 rpm for 20 min to remove insolubles; 1st, AAV purified with one round of CsCl ultracentrifugation; 2nd, AAV purified with two rounds of CsCl ultracentrifugation. (A) AAV5-CMV-luciferase purified from 3 L of Sf9 cell culture; (B) AAV5-CMV-GFP purified from 3 L of Sf9 cell culture; and (C) AAVDJ-CMV-GOI purified from 25 L of Sf9 cell culture. Error bar indicates the SD.

After all conditions of the TPP method were established, we performed three batches of large-scale AAV manufacturing and purification with the TPP process followed by one or two rounds of CsCl ultracentrifugation. The results are shown in Figure 5. The first batch was AAV5-cytomegalovirus (CMV)-luciferase vectors with a genome size of 3,317 bases. As shown in the results, 78% of AAV5 vectors were recovered at the end of the TPP process (compare interphase supernatant [Int-sup] with Lysate in Figure 5A), indicating that there was minimal loss of AAV vectors during the TPP process. When the bulk of AAV vectors were subjected to one round of CsCl ultracentrifugation in two SW28 centrifuge tubes, a total of 3.75e+15 vg of purified AAV5 vectors were obtained from this 3-L production run, with an average yield of 1.25e+15 vg/L cell culture. The second batch was AAV5-CMV-GFP with a genome size of 2,544 bases. At the end of the TPP process, more than 70% of AAV vectors were recovered (Figure 5B, Int-sup). After one round of CsCl ultracentrifugation in two SW28 centrifuge tubes, a total of 5.55e+15 vg were purified from this 3-L production run, with an average yield of 1.85e+15 vg/L cell culture. For the third production run, a 25-L culture was used. This time a different serotype, AAVDJ-CMV-gene of interest (GOI) with genome size of 4,661 bases, was used. This 25-L production run was collected into ten 1-L bottles and each bottle was added with 300 mL of Sf9 lysis buffer to prepare lysate. The results are shown in Figure 5C. At the end of the TPP process, 240 mL of Int-sup was obtained and the titer was slightly higher than the lysate, which was apparently due to variation of quantitative real-time PCR quantification. The 240 mL of bulk AAV sample was divided into 10 SW28 tubes and subjected to CsCl ultracentrifugation at 28,000 rpm for 20 h. The AAV bands were collected and subjected to a second round of CsCl ultracentrifugation in 10 centrifugation tubes for 70.1 Ti rotor at 65,000 rpm for 20 h. A total of 5.09e+15 vg of vectors were purified, with an average yield of 2.04e+14 vg/L cell culture.

### Removal of Cellular and Baculoviral Impurities during the TPP-DGUC Purification Process

**Figure 6:**
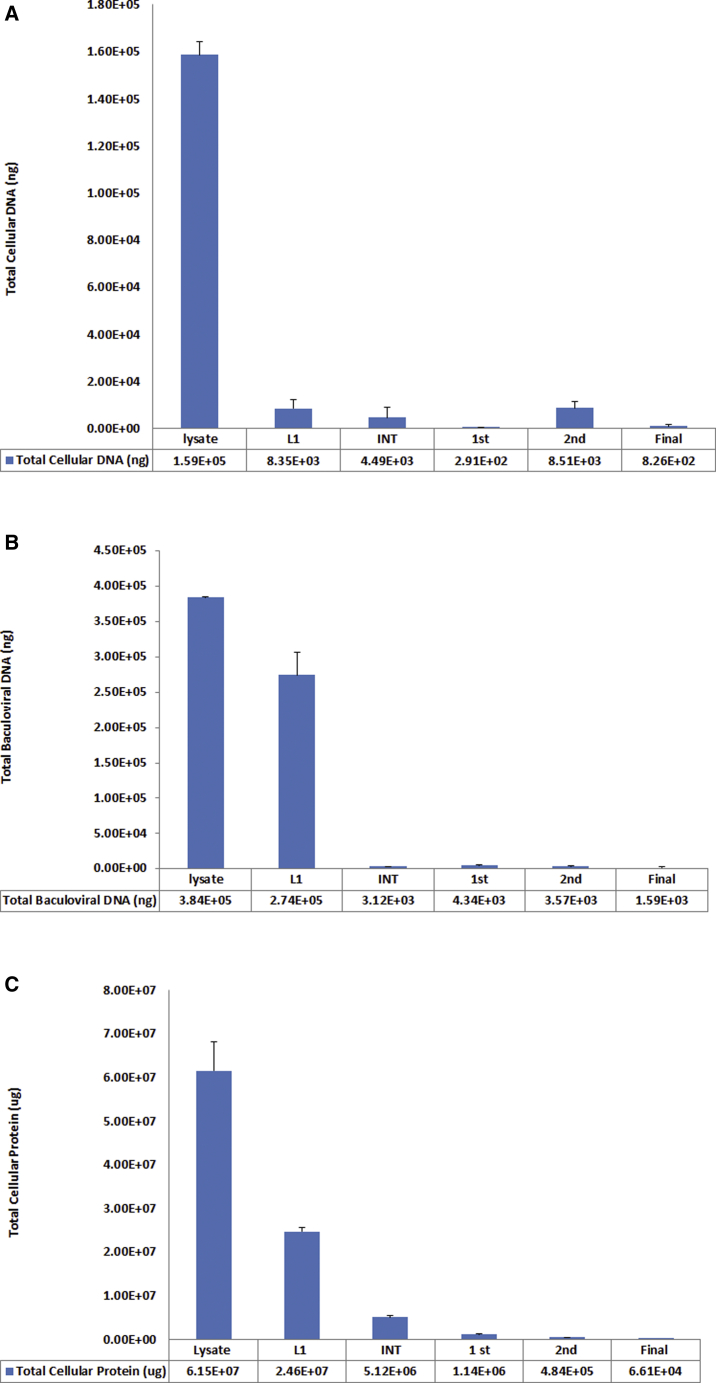
Removal of Impurities during the TPP-DGUC Process (A–C) Aliquots of samples from each step of the purification were collected and assayed with quantitative real-time PCR method to quantify the amounts of the cellular DNA (A), baculoviral DNA (B), and cellular proteins (C). Cell pellets from 25 L of Sf9 cell culture were collected in ten 1-L centrifugation bottles and centrifuged to remove the media. A total of 3,250 mL of cleared cell lysate was obtained and processed. Error bar indicates the SD.

Removal of impurities such as cellular proteins, genomic DNA, and baculoviral DNA is critical for the purification of AAV vectors. We employed quantitative real-time PCR assays to quantify these residual DNAs and bicinchoninic acid (BCA) assays to quantify the residual cellular proteins in each step of the TPP-DGUC process, and the results are shown in Figure 6. From these results we can see that the content of Sf9 cellular DNA was high in the cell lysate (159 μg of total cellular DNA in 3,250 mL of cleared lysate) but gradually decreased during the purification process (Figure 6A). In the final AAV sample there was only about 3.15 ng/mL Sf9 cellular DNA in the final AAV product at a titer of 2e+13 vg/mL (total of 826 ng of cellular DNA in 262 mL of AAV final product). There is a slightly higher amount of residual baculovirus DNA (6.08 ng/mL) in the final AAV product (Figure 6B). The removal of most cellular proteins (∼90%) during the TPP process has been shown during the optimization processes (Figures 1, 2, and 3). We further performed BCA assays to follow the decrease of total cellular proteins during the purification process, and the results are shown in Figure 6C. Total cellular protein content was high in the cleared cell lysate (61.5 g in 3,250 mL of cell lysate) and gradually decreased to about 252 μg/mL in the final AAV product.

### Comparison of Infectivity between TPP-Purified and DGUC-Purified AAV Vectors

Since the TPP process used two chemical components (ammonium sulfate and *tert*-butanol) that were not present in the DGUC purification process, we sought to determine whether there was any detrimental impact on the AAV infectivity by these two components. Three pairs of AAV vectors (AAV2-CMV-GFP, AAV5-CMV-GFP, and AAV6-CMV-GFP), one purified by TPP only and the other by DGUC only, were used in the *in vitro* assays. The results are shown in Table 1. As the results show, the GFP expression between TPP- and DGUC-purified AAV vectors was very similar, indicating no detrimental impact on the AAV infectivity by the TPP purification process.

**Table 1:** Comparison of Infectivity of AAV Vectors Purified by TPP or CsCl Ultracentrifugation Methods

Sample	Description^a^	Green Intensity Value^b^	Infectivity Ratio^c^
1	AAV2-CMV-GFP, CsCl purified	71.57	0.84
2	AAV2-CMV-GFP, TPP purified	85.18	1.00
3	AAV5-CMV-GFP, CsCl purified	27.91	0.93
4	AAV5-CMV-GFP, TPP purified	29.87	1.00
5	AAV6-CMV-GFP, CsCl purified	27.99	0.85
6	AAV6-CMV-GFP, TPP purified	33.06	1.00

a AAV vectors of 1.5e+9 vg/well were added to each well with HEK293T cells seeded at density of 1.5e+5 cells/well and incubated at 37°C in a CO_2_ incubator for 3 days.

b Green intensity value was measured on the whole optical field with free software ImageJ (NIH).

c The higher intensity value of each pair of AAV vectors was set to 1.00.

**Figure 7:**
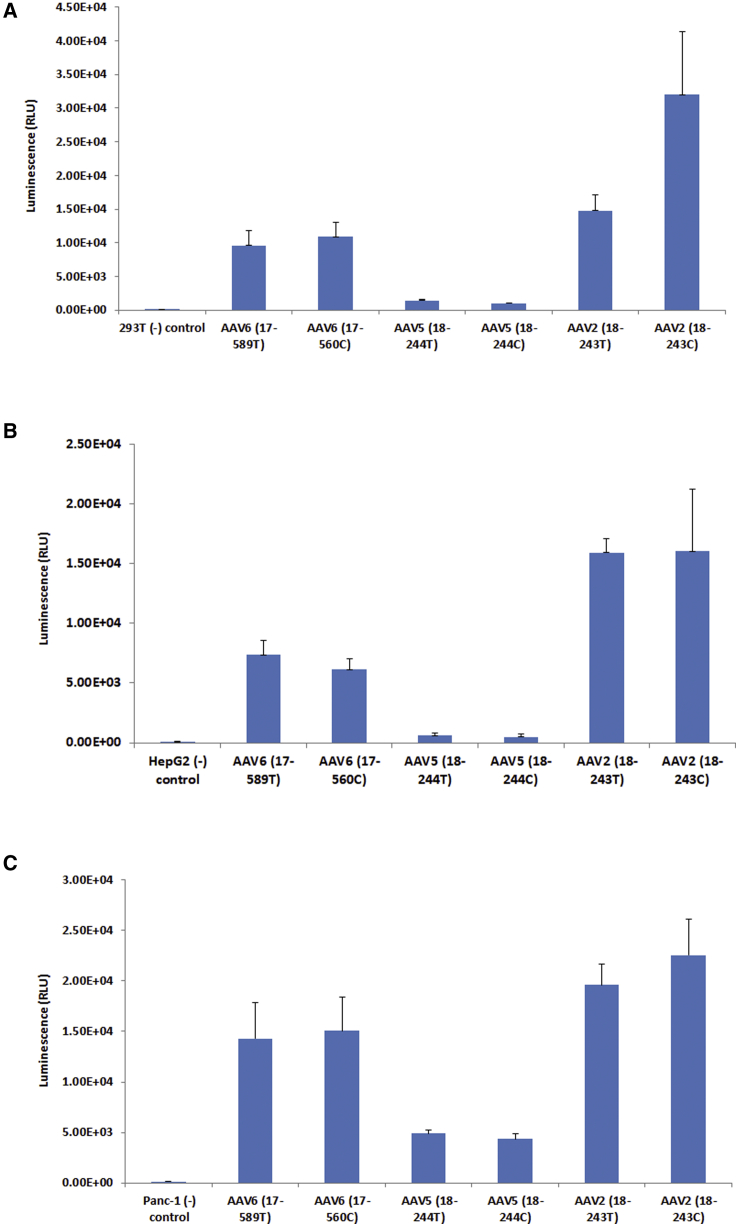
Comparison of AAV Infectivity with *In Vitro* Assays in Three Different Mammalian Cell Lines (A–C) The mammalian cells were seeded at a density of 3e+4 cells/well in 96-well plates overnight. The next morning old media were removed and 100 μL/well of AAV vectors was added. After overnight culture in a CO_2_ incubator at 37°C, 100 μL/well of media containing 20% FBS and antibiotics was added and the cells were transduced for a total of 72 h. Cell lysates were prepared and luciferase activities were recorded with the Tecan 384 plate reader. T, AAV purified with TPP-DGUC method; C, AAV purified with DGUC method. Error bar indicates the SD. (A) HEK293T cells. (B) HepG2 cells. (C) Panc-1 cells.

Because AAV vectors purified by the TPP process alone contain about 10% cellular impurities, which may impact the AAV infectivity either positively or negatively, we needed to further purify the AAV vectors with DGUC to remove the remaining 10% impurities. In order to know whether this combined TPP-DGUC purification process has any impact on AAV infectivity, we produced three AAV vectors (AAV2, AAV6, and AAV9-CMV-luciferase) and respectively purified them with DGUC or TPP-DGUC to compare the AAV infectivity. These AAV vectors were transduced into three different mammalian cell lines, and luciferase expression was measured. The results are shown in Figure 7. When the AAV infectivity was assayed with HEK293 cells, AAV2 purified by DGUC alone appeared to have higher infectivity than did TPP-DGUC-purified AAV2, whereas there was no infectivity difference for AAV5 and AAV6 vectors either purified with DGUC or with the TPP-DGUC method (Figure 7A). When AAV infectivity was assayed with HepG2 or Pac-1 cell lines, there was essentially no difference in infectivity between DGUC- and TPP-DGUC-purified AAV vectors (Figures 7B and 7C). These results indicate that the TPP-DGUC purification process does not have a detrimental impact on AAV infectivity.

**Figure 8:**
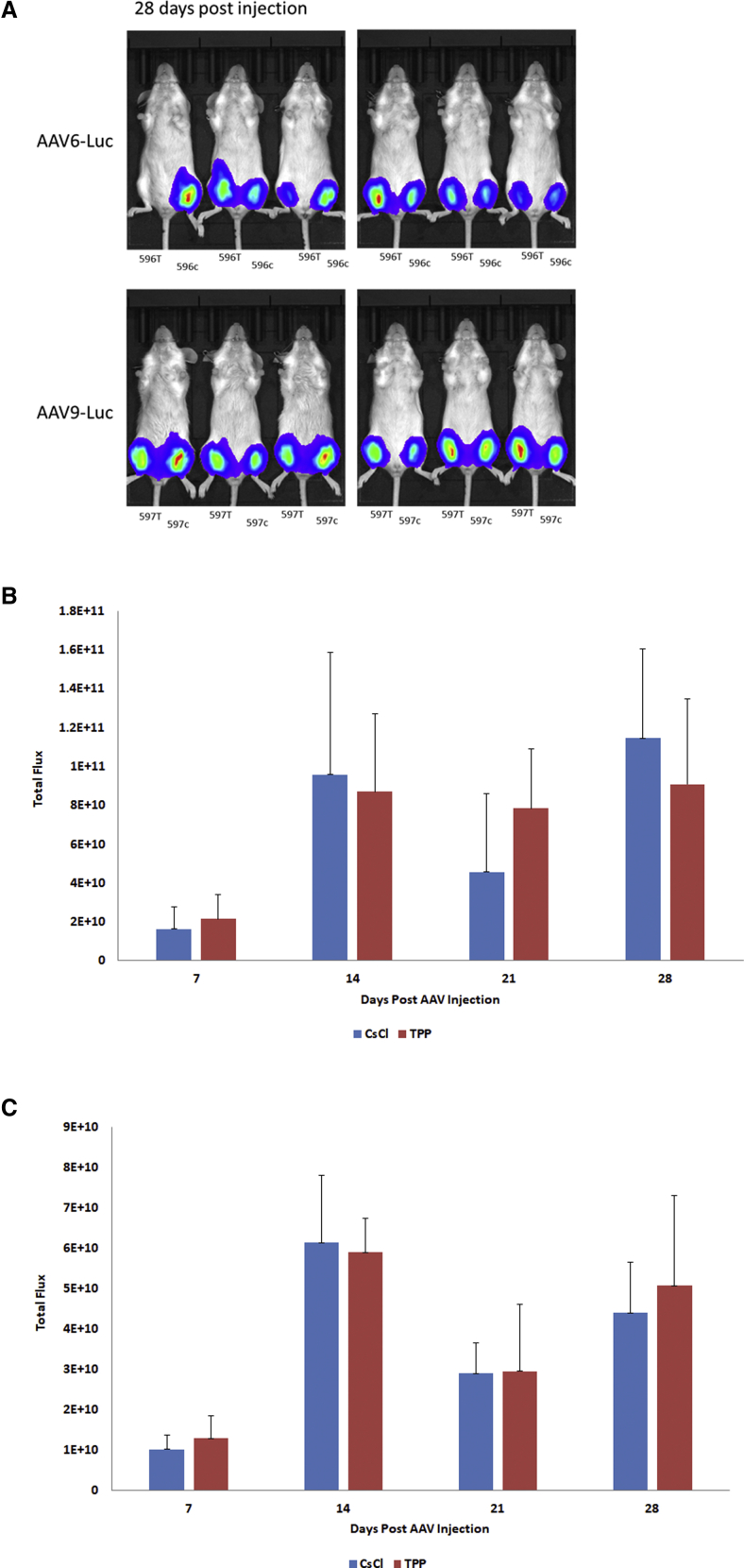
Comparison of AAV Infectivity with *In Vivo* Animal Studies (A–C) BALB/c nude mice were randomly divided into two groups, one for AAV6-CMV-luciferase and the other for AAV9-CMV-luciferase. In each group, animals were injected with 30 μL of DGUC-purified AAV vectors into the left gastrocnemius of hindleg and 30 μL of TPP-DGUC-purified AAV vectors into the right gastrocnemius of hindleg with the same titer. Bioluminescence was measured at days 7, 14, 21, and 28 after injection and images were recorded with an IVIS 100 imaging system. T, AAV purified with TPP-DGUC method; C, AAV purified with DGUC method. Error bar indicates the SD. (A) A representative image recorded at 28 days after injection. (B) AAV6-CMV-luciferase activities. (C) AAV9-CMV-luciferase activities.

We also wanted to know if the TPP-DGUC process would impact AAV infectivity in animal studies. To minimize the potential variation between production lots, this time we produced one lot of AAV6-CMV-luciferase and one lot of AAV9-CMV-luciferase and purified half of each with DGUC and the other half of each with TPP-DGUC process. All AAV vectors tested as endotoxin negative (data not shown), and each mouse was injected with an equal amount of AAV vectors purified by the two methods, one in the right leg and the other in the left leg. The results are shown in Figure 8. As the results show, both AAV6 and AAV9 vectors showed very similar luciferase expression regardless of the purification methods, demonstrating that TPP has no detrimental effect on *in vivo* AAV infectivity.

## Discussion

Development of an economical and universal purification process that can separate empty from full virus particles regardless of AAV serotypes will bring great advantages to gene therapy research. It has been reported that empty capsids are detrimental to gene therapy because they are a source of unnecessary and potentially antigenic materials, which could possibly induce or trigger anti-AAV innate and adaptive immune responses.^13^^,^^14^ Several groups have reported methodologies of AAV purification that are able to separate empty from full particles. One of the methods was using polyethylene glycol precipitation to condense AAV vectors followed by CsCl ultracentrifugation to obtain AAV vectors devoid of empty capsids.^15^ However, this method was limited by the fact that the polyethylene glycol-precipitated AAV sample still contained a significant amount of cellular impurities and therefore was suitable only for small-scale AAV manufacturing. A second method described using column chromatography to capture AAV vectors followed by CsCl ultracentrifugation to remove empty capsids, and the AAV vectors purified by this method were used in clinical studies.^16^ The limitation of this method is that each AAV serotype requires a specific resin/process and it is not universal. A third method described using cation exchange chromatography to separate AAV2 vectors from empty capsids.^17^ This method could recover 74% of the input AAV2 vectors but still contained more than 20% of empty capsids.

**Figure 9:**
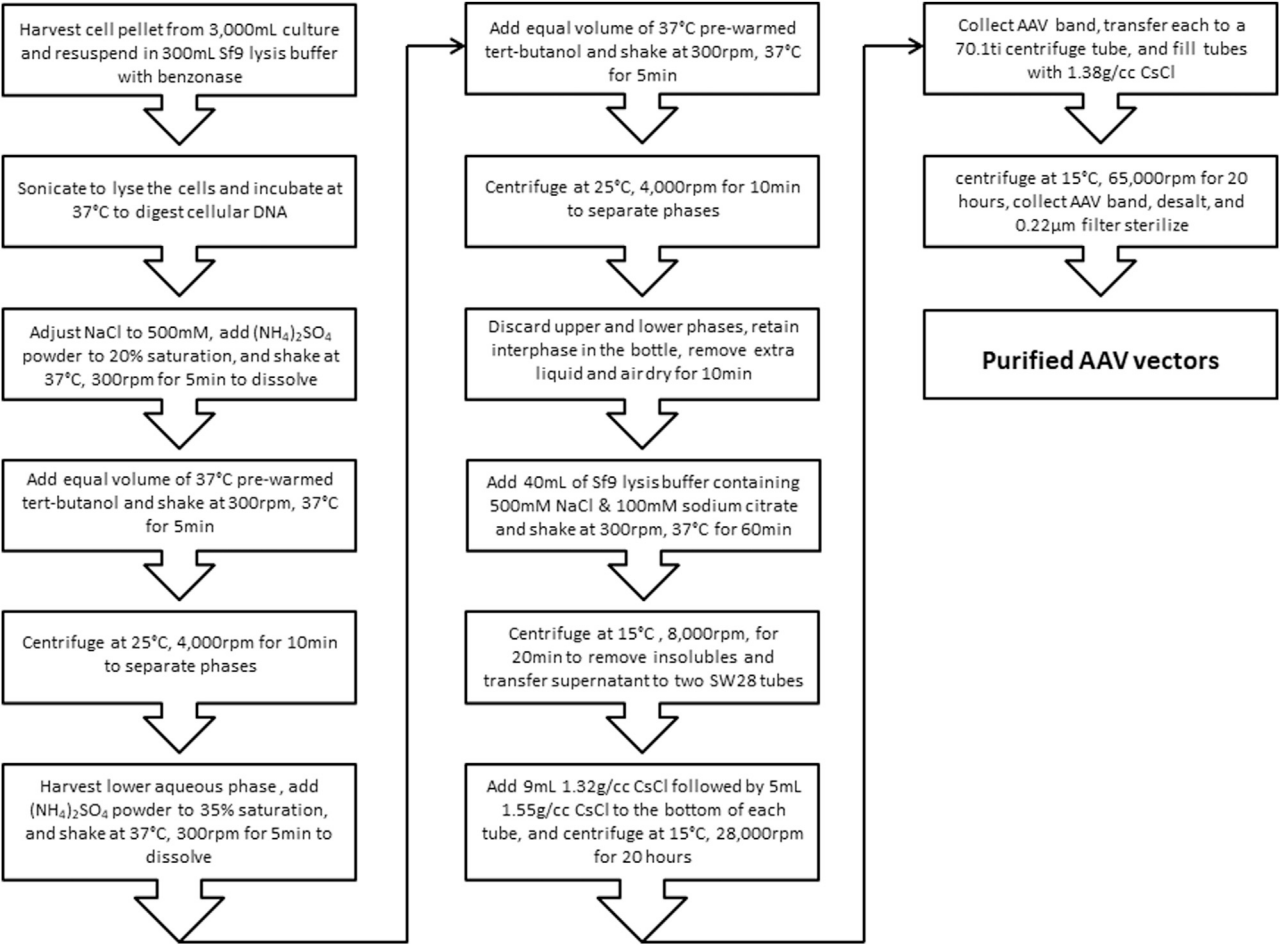
Flowchart of TPP-DGUC Purification Process

TPP has been used as an economical and nonchromatographic method to separate bioactive molecules from natural sources.^18^, ^19^, ^20^, ^21^, ^22^ However, TPP has never been used for any virus purification. In the present study, we applied the TPP process to purify AAV vectors. Initially, we used only one round of TPP with various combinations of ammonium sulfate concentrations and *t*-butanol ratio but failed to separate AAV particles from the cellular proteins. The AAV particles either remained in the aqueous phase at lower concentrations of ammonium sulfate or they were salted out together with cellular proteins at higher concentrations of ammonium sulfate. Based on this observation, we used a two-round TPP approach. In the first round of TPP, we gradually increased the concentration of ammonium sulfate to a certain value at which cellular proteins were salted out as much as possible but AAV particles were kept in the aqueous phase. To this aqueous phase we performed a second round of TPP by gradually increasing the concentration of ammonium sulfate to a certain value at which AAV particles were salted out but the remaining cellular proteins were kept in the aqueous phase. With these two-round TPP approaches, we developed the optimal conditions to purify bulk AAV vectors with 90% of cellular proteins removed. This process has been tested for AAV2, AAV5, AAV6, AAV9, and AAVDJ with equal efficiency and should be applicable to all serotypes of AAV. This process is significant since it is able to remove 90% of the cellular proteins and impurities and decrease the cell lysate volume 5- to 10-fold depending on the AAV titers in the cell lysates. Based on our experimental results, each SW28 centrifuge tube could hold as much lysate as prepared from 1.5-L cell culture for high-yield AAV serotypes such as AAV2, AAV5, and AAV6. For low-yield AAV serotypes such as AAVDJ, each SW28 centrifuge tube could hold as much lysate as prepared from 3-L cell culture without compromising the separation of empty virus from the full virus particles. This TPP process provides a tremendous advantage for the downstream process. We combined it with two rounds of CsCl ultracentrifugation and were able to purify more than 3e+15 vg of AAV particles devoid of empty capsids in just two SW28 centrifuge tubes (Figures 5A and 5B). By this calculation, a single Beckman Coulter or equivalent ultracentrifuge with six SW28 centrifuge tubes will be able to purify more than 1e+16 vg of AAV vectors that will provide enough materials for a small-scale clinical trial. Based on the data we obtained, we devised a detailed step-by-step flowchart as shown in Figure 9. This flowchart should provide good guidance for anyone who has interest in utilizing this TPP-DGUC process for AAV vector purification.

The cost of gene therapy drugs is extremely high partly due to the costs of manufacturing goods. Commonly used column chromatography resin AVB Sepharose High Performance (HP) costs about US$50/mL,^23^ and its AAV binding capacity is in the range of 1e+12 vg/mL according to the manufacturer’s description (GE Healthcare). To purify 1e+17 vg of AAV vectors it would use a 100-L volume of AVB Sepharose HP and the cost would be US$5,000,000, whereas the costs of materials for the process of TPP-DGUC would be less than US$2,000. This would bring down the cost of goods substantially and make gene therapy drugs more affordable. Furthermore, the empty capsids co-purified with AVB or other resins are not removed, whereas this TPP-DGUC combination process is able to purify any AAV serotypes devoid of the empty capsids. Therefore this novel process provides an economical and universal method for purification of AAV vectors in the range of 10^17^-vg scale. We fully aware that there is always a limitation to the centrifugation method and that purification of AAV vectors in the 10^18^-vg scale would require multiple purification runs with this process. A better and more scalable method that can meet the demand of such a large quantity of AAV product is still needed.

## Materials and Methods

### Sf9 Cell Culture

Sf9 cells were cultured in Corning storage bottles at 28°C in ESF 921 or ESF AF medium (Expression Systems, Davis, CA, USA) supplemented with 100 U/mL penicillin and 100 μg/mL streptomycin (Mediatech, Manassas, VA, USA). The cells were split 1:4 for maintenance once the cell density reached about 1e+7 cells/mL.

### Mammalian (HEK293, Panc-1, and HepG2) Cell Culture

HEK293, Panc-1, and HepG2 cells were cultured in T-75 flasks in a CO_2_ incubator at 37°C in DMEM media supplemented with 100 U/mL penicillin and 100 μg/mL streptomycin (Mediatech) and 10% fetal bovine serum (FBS) (ATCC, Manassas, VA, USA). The cells were split 1:10 for maintenance once they reached confluence.

### Production of AAV Vectors

AAV vector production was performed according to Virovek’s protocol. Briefly, Sf9 cells were cultured to about 1e+7 cells/mL and diluted 1:1 with fresh ESF 921 or ESF AF media. The diluted Sf9 culture was double infected with 10 MOIs of recombinant baculovirus (rBV) carrying the designated rep-cap genes and 5 MOIs of rBV containing the GOI. For small-scale production, Sf9 cells of 200- to 300-mL volume in 1-L Corning storage bottles were infected at 28°C, 160 rpm in a shaker incubator for 3 days. For large-scale production, Sf9 cells in a 25/50 Wave bioreactor system were culture in a 12-L volume until reaching a density of ∼1e+7 cells/mL, and then 10 MOIs of rBV-rep-cap and 5 MOIs of rBV-GOI were added, followed by 12 L of fresh media. The infection was carried out at 25 rpm, 28°C for 3 days supplemented with 30% oxygen. The infected Sf9 cells were harvested by centrifugation at 3,000 rpm for 20 min, and cell pellets were used for cell lysate preparation or stored at −20°C if not used immediately.

### Preparation of Cell Lysate

Each AAV-containing cell pellet harvested from 200 to 300 mL of culture was lysed in 30 mL (for the TPP process) or 15 mL (for the DGUC process) of Sf9 lysis buffer (50 mM Tris-HCl [pH 7.8], 2 mM MgCl_2_, 1% sodium lauroyl sarcosinate [sarkosyl], and 1% Triton X-100) with a total of 3,750 U of Benzonase (EMD Millipore, Billerica, MA, USA) by sonicating for 30 s. Genomic DNA was digested by incubation at 37°C for 1 h. After digestion, the cell lysates were adjusted to ∼500 mM NaCl. For the large-scale production run, a cell pellet collected from 3 L of culture was lysed in 300 mL of Sf9 lysis buffer for the TPP process with sonication and processed the same way as described above.

### Three-Phase Partitioning

Ammonium sulfate powder (Fisher Scientific, Princeton, NJ, USA) was added to the cell lysate to the desired concentration values and dissolved by shaking at 300 rpm, 37°C for 5 min in a shaker incubator. The concentration values were calculated according to an ammonium sulfate calculator (EnCor Biotechnology, Gainesville, FL, USA). An equal volume of *tert*-butanol (Acros Organics, Princeton, NJ, USA) pre-warmed at 37°C was then added to the cell lysate and mixed through vigorous shaking at 300 rpm, 37°C for 5 min in the shaker incubator. The mixture was then centrifuged at 4,000 rpm for 10 min at room temperature to form three phases (upper phase, interphase, and lower phase). After removal of the upper phase, the AAV-containing first lower aqueous phase (L1) was collected and the interphase containing a majority of cellular proteins, lipids, and other cellular debris was discarded. This completed the first round of TPP. To the collected aqueous phase L1, additional ammonium sulfate was added to the desired concentration values and dissolved by shaking at 300 rpm, 37°C for 5 min. Then, an equal volume of *t*-butanol was added and mixed by vigorously shaking at 300 rpm, 37°C for 5 min in the shaker incubator. The mixture was centrifuged at 4,000 rpm for 10 min at room temperature to form three phases. The upper phase was removed and the second lower aqueous phase (L2) was collected for determination of the remaining AAV vectors. The AAV-containing solid interphase was briefly dried to evaporate the *t*-butanol and fully dissolved in 20–40 mL of Sf9 lysis buffer containing 500 mM NaCl and 100 mM sodium citrate by shaking at 300 rpm, 37°C for 60 min. The dissolved interphase (Int) was then centrifuged at 8,000 rpm for 20 min to remove insoluble debris and the clear supernatant (Int-sup) was collected. For assays to determine AAV infectivity purified by the TPP process in mammalian cells, the Int-sup was buffer exchanged and sterile filtered. Otherwise, it was subjected to one or two rounds of DGUC with CsCl media for further purification.

### Three-Phase Partitioning Combined with DGUC

After the TPP process and before buffer exchange, the bulk AAV samples were further purified with DGUC to remove empty capsids as well as the remaining impurities. Briefly, ∼23 mL of TPP-processed AAV samples was loaded in one ultraclear centrifuge tube for SW28 rotor followed by 9 mL of 1.32 g/cc and 5 mL of 1.55 g/cc CsCl solutions and centrifuged at 28,000 rpm, 15°C for 18–20 h. At the end of centrifugation, the centrifuge tubes were assembled on a stand and AAV bands were visualized with a beam light shining underneath the tube. A syringe needle of 18G was inserted about 0.5 cm below the full AAV band to slowly collect 5–8 mL of AAV sample depending on the intensity of the AAV band. An AAV empty band was left in the tube or could be collected for analysis if needed. In some experiments, these AAV samples were subjected to a second round of DGUC by mixing with CsCl solution of 1.38 g/cc in the ultraclear centrifuge tubes for a 70.1 Ti or 50.2 Ti rotor. After being heat sealed, the tubes were balanced and centrifuged at 65,000 or 50,000 rpm, 15°C for 18–20 h. At the end of centrifugation, the tubes were assembled on a stand with a beam light shining underneath the tube to visualize the AAV band. A syringe needle of 18G was inserted about 0.5 cm below the full AAV band to slowly collect 2–5 mL of AAV sample. After buffer exchange and sterile filtration, the AAV samples were assayed with quantitative real-time PCR to determine the quantity, and with SDS-PAGE followed by SimplyBlue SafeStain (Invitrogen) to verify the purity.

### SDS-PAGE and Protein Staining

The AAV vectors were mixed with SDS-PAGE loading buffer (Invitrogen) and heated at 95°C for 5 min. Then, they were loaded onto 10% SDS-PAGE gels and run at 100 V until the dye reached the bottom of the gels. The gels were stained with SimplyBlue SafeStain (Invitrogen) according to the manufacturer’s protocol. The gel images were scanned and recorded.

### Measurement of Protein Concentration

Protein concentrations in cell lysates and samples at different purification steps were measured according to manufacturer’s protocol (Thermo Fischer Scientific, CA, USA). Briefly, protein samples were diluted in sterile water to a desired dilution factor. Then, each 25 μL of samples was mixed with 200 μL of BCA working reagent solution (prepared fresh by mixing at 50:1 ratio of reagent A and reagent B). After incubation at 37°C for 15–20 min, optical density (OD) values were recorded in a Tecan Ultra384 plate reader (Tecan Schweiz, Mannedorf, Switzerland) and protein contents were calculated according to the standards.

### Quantification of AAV Vectors

Quantities of AAV particles were determined using a quantitative real-time PCR assay in accordance with the manufacturer’s protocol (Bio-Rad, Hercules, CA, USA). Briefly, AAV samples were diluted to a suitable range in quantitative real-time PCR dilution buffer (10 mM Tris-HCl [pH 8.0], 1 mM EDTA, 10 μg/mL yeast tRNA, 0.01% Tween 80) and 10 μL of the diluted AAV samples was mixed with 39 μL of DNaseI digestion buffer (10mM Tris-HCl [pH 7.6], 2.5 mM MgCl_2_, 0.5 mM CaCl_2_) and 1 μL (2 U) of DNaseI (New England Biolabs). A National Reference Standard Stock (RSS) rAAV2-RSS (ATCC) was included in all quantitative real-time PCR assays as an internal control. The digestion was carried out for 1 h at 37°C to remove contaminating DNA from the surface of the virus particles. At the end of digestion, the AAV samples were mixed with an equal volume of 200 mM EDTA and heated to 95°C for 30 min to inactivate the DNaseI. The digested AAV samples were further diluted from 1:100 to 1:1,000 with quantitative real-time PCR dilution buffer and used for a quantitative real-time PCR assay in the Chromo4 real-time detector with primers/probe set corresponding to the AAV2 inverted terminal repeats (ITRs) (forward primer, 5′-GGAACCCCTAGTGATGGAGTT-3′; reverse primer, 5′-CGGCCTCAGTGAGCGA-3′; probe, 5′-FAM-CACTCCCTCTCTGCGCGCTCG-MGB-3′).^24^

### Quantification of Residual Baculoviral and Sf9 Cellular DNAs

Both baculoviral and Sf9 cellular residual DNAs were quantified with the quantitative real-time PCR method. To quantify the baculoviral residual DNA in the AAV samples, a set of primers/probe (forward primer, 5′-TCGGTGCTCGACTTTGCGTT-3′; reverse primer, 5′-GAGTCGGTGACACGCGAACA-3′; probe, 5′-FAM-TGCATCTGTTAAACTTGCAGTTCCACG-MGB-3′) corresponding to the baculoviral gp64 gene was used. The quantity of residual baculoviral DNA was calculated based on the standard curve of purified baculoviral DNA assayed at the same time. To quantify the residual Sf9 cellular DNA, a set of primers/probe (forward primer, 5′-ACATCACTCAGTCCGCAGGT-3′; reverse primer, 5′-TCCTCAATCTTGGGTGCTAGGT-3′; probe, 5′-FAM-GCCGACGTACCACTTGTCGTCG-MGB-3′) corresponding to the Sf9 housekeeping gene proliferating cell nuclear antigen (PCNA) was used. The quantity of residual Sf9 cellular genomic DNA was calculated based on the standard curve of purified Sf9 genomic DNA assayed at the same time. Briefly, the samples were diluted to a suitable range (from 1:100 to 1:1,000) in quantitative real-time PCR dilution buffer, and 10 μL of each diluted sample was used for quantitative real-time PCR assays in the Chromo4 real-time detector with the corresponding primers/probe set.

### Endotoxin Assay

Endotoxin levels in the purified AAV samples were measured with the Limulus Amebocyte Lysate (LAL) PYROGENT 06 Plus kit (Lonza) according to the manufacturer’s protocol. Briefly, purified AAV samples were diluted 1:10 in endotoxin-free water, and diluted AAV samples (100 μL) were each mixed in glass tubes with 100 μL of reconstituted LAL by gentle vortexing for 5 s. After incubating at 37°C for 1 h, the tubes were carefully removed and inverted at 180°C. A positive reaction was indicated by the formation of a firm gel that remained intact momentarily when the tube was inverted. A negative reaction was indicated by the absence of a solid clot after inversion. The endotoxin level was calculated based on the dilution factors.

### *In Vitro* Assay of GFP Expression

Purified AAV vectors were used to transduce HEK293T cells to determine their infectivity. Briefly, HEK293T cells were seeded at 1.5e+5 cells/well with 0.5 mL of DMEM media containing 10% FBS and 100 U/mL penicillin and 100 μg/mL streptomycin in 24-well plates and cultured at 37°C in a CO_2_ incubator overnight. The next morning a 10-fold serial dilution of the AAV samples was prepared in culture media without FBS but containing 20 μM etoposide. After removing the old media, 0.5 mL of the diluted AAV samples was added to each well and the cells were incubated in the CO_2_ incubator at 37°C overnight. The next morning, 0.5 mL of DMEM media containing 20% FBS and 100 U/mL penicillin and 100 μg/mL streptomycin was added and cells were cultured for a total of 72 h. GFP-expressing cells were photographed, and the intensity of green color was determined with the free software ImageJ (NIH).

### *In Vitro* Assay of Luciferase Activity

Mammalian cells (HEK293T, HepG2, and Panc-1) were cultured in T-75 flasks in DMEM media containing 10% FBS and 100 U/mL penicillin and 100 μg/mL streptomycin until 70%–90% were confluent in a CO_2_ incubator at 37°C. The cells were trypsinized and plated in a 96-well plate at 3e+4 cells/well in a 100-μL vol and cultured overnight. The next morning, purified AAV vectors were diluted in serum-free DMEM containing 20 μM etoposide. After removing the old media from the 96-well plate, 100 μL/well of the diluted AAV vectors was added. After overnight culture, another 100 μL/well of DMEM containing 20% FBS and 100 U/mL penicillin and 100 μg/mL streptomycin was added and the cells were transduced for a total of 72 h at 37°C in the CO_2_ incubator. The luciferase activity was assayed with a Pierce firefly luciferase glow assay kit according to the manufacturer’s protocol (Thermo Fisher Scientific). Briefly, the transduced cells were lysed in 50 μL/well of lysis buffer with gentle shaking for 15–30 min at room temperature. Twenty microliters of lysate was mixed with 50 μL of working solution in a white opaque 96-well plate. After incubation for 10 min, the luminescence signal was recorded in a Tecan Ultra 384 plate reader.

### *In Vivo* Assay of AAV Infectivity

All animal procedures were performed with prior approval of the Institutional Animal Care and Use Committee (IACUC) at JOINN Laboratories CA. BALB/c nude mice (female, 6–8 weeks of age, weighing approximately 17–22 g) were used for the study. Briefly, 12 animals were randomly divided into two groups, one for AAV6-CMV-luciferase and the other for AAV9-CMV-luciferase. In each group, animals were injected with 30 μL of DGUC-purified AAV vectors into the left gastrocnemius of the hindleg and 30 μL of TPP-DGUC-purified AAV vectors into the right gastrocnemius of the hindleg. Both AAV preparations were diluted to the same titer at 1.5e+13 vg/mL based on the quantitative real-time PCR titration method before injection. For each animal, body weight was monitored every week and bioluminescence was measured at days 7, 14, 21, and 28 after injection. Bioluminescence imaging was performed with an IVIS 100 imaging system equipped with a camera box and warming stage, and data were recorded.

## Author Contributions

Z.Y. conducted TPP experiments, S.Z. and C.Y.H. conducted AAV production and purifications, N.L. conducted quantitative real-time PCR assays, M.C. conducted animal study, and H.C. conceived the TPP idea and wrote the paper.
